# The Resident Assessment Instrument-Minimum Data Set 2.0 quality indicators: a systematic review

**DOI:** 10.1186/1472-6963-10-166

**Published:** 2010-06-16

**Authors:** Alison M Hutchinson, Doris L Milke, Suzanne Maisey, Cynthia Johnson, Janet E Squires, Gary Teare, Carole A Estabrooks

**Affiliations:** 1 School of Nursing and Midwifery, Deakin University, and Cabrini-Deakin Centre for Nursing Research, Cabrini Institute, Cabrini Health, Melbourne, Victoria, Australia; 2 CapitalCare Edmonton Area and Faculty of Rehabilitation Medicine, Faculty of Nursing, and Department of Psychology, University of Alberta, Edmonton, Alberta, Canada; 3 Shepherd's Care Foundation, Edmonton, Alberta, Canada; 4 Health Professions Strategy and Practice, Alberta Health Services, Edmonton, Alberta, Canada; 5 Knowledge Utilization Studies Program (KUSP), Faculty of Nursing, University of Alberta, Edmonton, Alberta, Canada; 6 Saskatchewan Health Quality Council, Saskatoon, Saskatchewan, Canada

## Abstract

**Background:**

The Resident Assessment Instrument-Minimum Data Set (RAI-MDS) 2.0 is designed to collect the minimum amount of data to guide care planning and monitoring for residents in long-term care settings. These data have been used to compute indicators of care quality. Use of the quality indicators to inform quality improvement initiatives is contingent upon the validity and reliability of the indicators. The purpose of this review was to systematically examine published and grey research reports in order to assess the state of the science regarding the validity and reliability of the RAI-MDS 2.0 Quality Indicators (QIs).

**Methods:**

We systematically reviewed the evidence for the validity and reliability of the RAI-MDS 2.0 QIs. A comprehensive literature search identified relevant original research published, in English, prior to December 2008. Fourteen articles and one report examining the validity and/or reliability of the RAI-MDS 2.0 QIs were included.

**Results:**

The studies fell into two broad categories, those that examined individual quality indicators and those that examined multiple indicators. All studies were conducted in the United States and included from one to a total of 209 facilities. The number of residents included in the studies ranged from 109 to 5758. One study conducted under research conditions examined 38 chronic care QIs, of which strong evidence for the validity of 12 of the QIs was found. In response to these findings, the 12 QIs were recommended for public reporting purposes. However, a number of observational studies (n = 13), conducted in "real world" conditions, have tested the validity and/or reliability of individual QIs, with mixed results. Ten QIs have been studied in this manner, including falls, depression, depression without treatment, urinary incontinence, urinary tract infections, weight loss, bedfast, restraint, pressure ulcer, and pain. These studies have revealed the potential for systematic bias in reporting, with under-reporting of some indicators and over-reporting of others.

**Conclusion:**

Evidence for the reliability and validity of the RAI-MDS QIs remains inconclusive. The QIs provide a useful tool for quality monitoring and to inform quality improvement programs and initiatives. However, caution should be exercised when interpreting the QI results and other sources of evidence of the quality of care processes should be considered in conjunction with QI results.

## Background

The Resident Assessment Instrument Minimum Data Set Version 2.0 (RAI-MDS 2.0) is a comprehensive, standardized tool to assess residents in long-term care (LTC) settings. Assessment with this instrument enables detection of residents' strengths, needs and potential risks to inform individualized care planning and monitoring. Typically, data collected from residents in a facility is aggregated to produce indicators of the quality of care provided (i.e., quality indicators, QIs) at an individual and at a facility level. Given the capacity for the QIs to flag potential quality problems and inform quality improvement programs, evidence for, and confidence in, the reliability and validity of the quality measures is of fundamental importance to current and potential residents of LTC facilities, healthcare providers, decision- and policy-makers, and researchers. The RAI-MDS 2.0 has been adopted in several countries and others are in the process of implementing this instrument. This review was initiated because of this heightened interest in the tool. The purpose of this review was to systematically examine published and grey research reports in order to assess the state of the science regarding the validity and reliability of the RAI-MDS 2.0 QIs.

Although the RAI-MDS was not originally designed as a quality measurement instrument, researchers have used RAI-MDS data elements to derive QIs [1]. These indicators have been systematically developed and subsequently tested to reflect quality of care processes and outcomes, and provide a basis for quality improvement programs in LTC settings [2,3]. QIs are calculated according to the presence or absence of a particular indicator for an individual. This data can then be summed for all individuals in a facility to provide a facility-level estimate for the occurrence of the QI [4]. Some indicators, such as *bedfast residents*, are computed according to their prevalence (i.e., number of existing occurrences), while others, such as *new fractures*, are calculated according to their incidence (i.e., number of new occurrences). The indicators "are not absolute measures of quality but are markers of potentially poor (or good) care practices and resident outcomes" [[5] p. 603]. Furthermore, addressing quality of care using the QIs requires that the indicators are valid and reliable [6].

Validity refers to the extent to which a measure achieves the purpose for which it is intended and is determined by the "degree to which evidence and theory support the interpretations of test scores entailed by proposed users of tests [[7], p. 9]. Validity is, therefore, the most fundamental consideration in evaluating scores obtained from any instrument. The type of validity information to be obtained depends on the aims of the measure. In the case of the RAI-MDS QIs, the aim is to provide indicators of potentially good or poor practice and, hence, the type of validity data that is sought reflects the quality of practice and specifically, resident outcomes. Reliability refers to the consistency of measurement obtained when using an instrument repeatedly on a population of individuals or groups [7]. Inter-rater reliability is often measured using the kappa statistic, a quantitative measure of the magnitude of agreement between two raters or the consistency between test results. A score of 1 represents 100% agreement and a score of zero indicates the extent of agreement is no better than that which would have occurred by chance. The level of agreement is often judged as follows: ≤20 = Poor, .21-.40 = Fair, .41-.60 = Moderate, .61-.80 = Good, ≥.81 = Very good [8].

The QIs provide a practical instrument for facilities to track quality of care over time, both at a resident and facility level [3]. The identification of potential problems can prompt the implementation of quality initiatives as a preventative measure, or in the event that a quality issue arises, corrective measures can be implemented. National benchmarking and within-facility comparisons can also be undertaken to detect changes in care quality in response to the implementation of quality initiatives [6].

### Origin and development of the RAI-MDS instrument

Development of the Resident Assessment Instrument (RAI) Minimum Data Set (MDS) was prompted by LTC reforms endorsed by the United States (U.S.) government with passage of the Omnibus Budget Reconciliation Act (OBRA) in 1987. OBRA required that all nursing home residents undergo a comprehensive assessment on a regular basis - on admission to a facility, each quarter, and following a significant change in health or functional status [9]. An international consortium of researchers and clinicians from over 30 countries, known as the interRAI network (www.interrai.org), formed to promote and guide the use of the RAI-MDS instrument. The introduction of the instrument in 1991 "made it possible to construct uniform measures based upon common data characterizing all residents of all facilities" [[10] Background, ¶ 3]. In 1995 a revised version of the RAI-MDS, the RAI-MDS 2.0, was developed. A number of data elements from the previously tested instrument were retained, others were modified, and new items were added, resulting in over 400 data elements [11]. This revised version was found to have improved reliability [10-12] and was introduced in the U.S. in 1996. Since then interRAI have introduced additional assessment instruments, each of which is tailored to a specific healthcare setting, such as acute care, post-acute care ("short-stay in-patient care setting to receive supplemental rehabilitative and restorative services" [13]), home care, mental health, and palliative care. In addition, a more recent version of the LTC assessment instrument, the interRAI Long Term Care Facility (LTCF), and an adaption of the RAI-MDS 2.0, the MDS 3.0, have been released. At this point in time the interRAI LTCF instrument has not been widely implemented. The MDS 3.0 has been implemented in the U.S. only, while in other countries the RAI-MDS 2.0 continues to be the instrument of choice for collection of assessment data in LTC settings. In Canada, for example, all Canadian provinces will be using the RAI-MDS 2.0 for at least the next five years. The continued use of the RAI-MDS 2.0 and the respective QIs in most countries, at least for the foreseeable future, underpins the significance of understanding the validity and reliability of the RAI-MDS 2.0 QIs, hence the relevance of this review.

### Validity and reliability of the RAI-MDS data

Several studies have validated the data elements contained in the first (RAI-MDS) and second (RAI-MDS 2.0) versions of the instrument against standardized instruments measuring similar characteristics [14-18]. The RAI-MDS data elements have also been tested comprehensively for inter-rater reliability, prior to, and following implementation, in a range of LTC settings [9-12,19,20]. However, a few studies have cast doubt on the reliability of some RAI-MDS data elements. A study conducted in 2001 by Abt Associates, on behalf of the Health Care Financing Administration, found discrepancies in 67% of the RAI-MDS instrument data elements [10]. Investigation revealed that the variations were due to errors in data entry, i.e., miscoding into neighboring categories, and systematic bias was not evident. Although actual agreement rates for a number of data elements were reported to be poor, reliability was reported to be adequate when calculated using a weighted kappa statistic [10].

A large international study examining the reliability of items from five interRAI instruments, including the recently revised LTC assessment instrument (interRAI LTCF), has produced better results [21]. A mean kappa score of .74, indicating good agreement [8], was found for items contained in the interRAI LTCF instrument that are also common to other instruments within the interRAI suite, including the RAI-MDS 2.0. The mean kappa score for items that are unique to the interRAI LTCF instrument exceeded .60. While the interRAI LTCF instrument contains some items that have been revised since the RAI-MDS 2.0 version of the instrument, these findings add to the evidence for reliability of some assessments items used in the LTC setting.

While validity and reliability of the data elements used to derive the QIs are critically important, they do not guarantee the indicator itself is reliable [22]. The history of the development of the RAI-MDS QIs is described in the following section, including their evolution from a set of 175 QIs derived from the first version of the RAI-MDS to the current set of 24 indicators (Table 1), derived from the RAI-MDS 2.0, that have been used for public reporting.

**Table 1:** CMS Nursing Home Compare Publically Reported RAI-MDS 2.0 Quality Indicators

Domain	Quality Indicator
Accidents	Incidence of new fractures
	Prevalence of falls
	
Behavioral and emotional patterns	Prevalence of behavioral symptoms affecting others
	Prevalence of symptoms of depression
	Prevalence of symptoms of depression without antidepressant therapy
	
Clinical management	Use of nine or more different medications
	
Cognitive patterns	Incidence of cognitive impairment
	
Elimination and continence	Prevalence of bladder/bowel incontinence
	Prevalence of occasional bladder/bowel incontinence without a toileting plan
	Prevalence of indwelling catheters
	Prevalence of fecal impaction
	
Infection control	Prevalence of urinary tract infections
	
Nutrition and eating	Prevalence of weight loss
	Prevalence of tube feeding
	Prevalence of dehydration
	
Physical functioning	Prevalence of bedfast residents
	Incidence of decline in late-loss ADLs
	Incidence of decline in range of motion
	
Psychotropic drug use	Prevalence of antipsychotic use in the absence of psychotic and related conditions
	Prevalence of anti-anxiety/hypnotic use
	Prevalence of hypnotic use more than two times in last week
	
Quality of life	Prevalence of daily physical restraints
	Prevalence of little or no activity
	
Skin care	Prevalence of stage 1 - 4 pressure ulcers

### Development of the RAI-MDS quality indicators

The Health Care Financing Administration (HCFA) (now the Centers for Medicare and Medicaid Services [CMS]) contracted the Center for Health Systems Research and Analysis (CHSRA) at the University of Wisconsin-Madison to complete the Nursing Home Case Mix and Quality (NHCMQ) Demonstration Project (1989-1998). As part of this project, clinicians and researchers derived from the RAI-MDS a draft set of QIs "that signal the presence or absence of potentially poor care practices or outcomes" [[23] p. 53]. These indicators were reviewed extensively by interdisciplinary panels of experts resulting in the refinement of some indicators, removal of others and the addition of some new indicators [9]. This resulted in 175 indicators that were organized into 12 health care domains. Ongoing analyses to test clinical validity, feasibility, and usefulness, resulted in a refined set of 30 QIs that cover process and outcome indicators, including prevalence and incidence measures [1,23]. For the indicators to be derived from data collected using the RAI-MDS, they were subsequently reduced to a total of 24, covering 11 health care domains (Table 1) [1]. These indicators were considered to be sensitive enough to enable discrimination of quality and to be responsive to staff interventions to improve quality of care [24], and have been used for public reporting on the CMS Nursing Home Compare website (http://www.medicare.gov/NHCompare/).

### Initial testing of the RAI-MDS quality indicators

Researchers from the CHSRA at the University of Wisconsin-Madison conducted early validation studies of the QIs [25-27]. As part of the NHCMQ Demonstration Project a limited validation study was undertaken prior to implementation of the QIs [3,23]. This study was conducted in nine facilities in the U.S. and included testing of over half of the 30 QIs derived from the original version of the RAI-MDS [23]. The QI data were compared with independent assessments based on observation, chart review and interviews of staff, residents and family members. The findings of this study suggested the QIs had a high level of validity, with facility QI accuracy rates reported to range from 72% to 95% [1,23], and average accuracy reported as 79% [23]. It was concluded that the QIs in general were useful in identifying potential quality issues [1,3,23].

Rantz et al. [5] undertook a study to examine 14 of the QIs derived from 1994-1995 RAI-MDS version 1.0 data. A purposive sample of seven nursing homes identified as performing well on the 14 QIs and seven, which were performing poorly on those indicators, were selected. Data were collected using participant observation to identify care processes and activities performed in relation to outcomes detected by each of the QIs. These data revealed that all RAI-MDS QIs tested were able to discriminate between nursing homes that provided good and poor care quality. Independent measures of quality verified the level of quality in each facility; providing evidence that the RAI-MDS QIs were associated with the observed levels of care quality. Rantz et al. concluded, "QIs derived from MDS data can serve as a reasonable first step in determining what level of quality exists in a facility" [[5] p. 59].

Using RAI-MDS data collected in 1996, Karon, Sainfort and Zimmerman [28] examined, using correlation coefficients and kappa statistics, the stability of the 30 QIs across three quarters of data collected in two states in the U.S. Correlation coefficients for all 25 prevalence QIs were statistically significant. Twenty of these QIs had correlation coefficients of .8 or more, indicating that the change in prevalence of the QIs over time, within each facility, was minimal. Correlation coefficients for the incidence QIs were also statistically significant but were lower, ranging from .23 and .64. It was concluded that while the QIs were generally stable over time they were also sensitive enough to detect differences.

Since introduction of the RAI-MDS 2.0 in 1996 a number of studies have been conducted to evaluate the reliability and validity of the QIs associated with this version of the instrument. Most studies examined single indicators (n = 13), but one study examined multiple indicators. We conducted this review in order to examine and integrate the evidence for the reliability and validity of the RAI-MDS 2.0 QIs for the benefit of healthcare providers, decision- and policy-makers, and researchers who use the QIs to inform practice, education and research.

## Methods

### Search strategy

A comprehensive and systematic search was undertaken to retrieve literature relevant to the validity and reliability of the RAI-MDS 2.0 QIs. A health sciences librarian assisted in constructing and executing the search of relevant bibliographic databases (Table 2). The search terms used for the individual databases are reported in Table 2. In addition to the bibliographic database search, a search was conducted for grey literature; Google Scholar was used and numerous websites were searched, including:

◆ Abt Associates Inc.

◆ Agency for Healthcare Research and Quality (AHRQ)

◆ Arizona Department of Health Services

◆ Canadian Institute for Health Information (CIHI)

◆ Centers for Medicare & Medicaid Services (CMS)

◆ InterRAI home and international websites

◆ Manitoba Centre for Health Policy and Evaluation

◆ National Research and Development Centre for Welfare and Health (STAKES)

◆ Ontario Joint Policy and Planning Committee (JPCC)

◆ Ontario Ministry of Health (OMH)

◆ General Accounting Office, United States

**Table 2:** RAI-MDS Quality Indicator Search

Database	Platform	Date Searched	Results
MEDLINE--In-Process and other non-indexed citations; MEDLINE Daily; and MEDLINE 1950 to present	OVID	Nov 27, 2008	686
EMBASE 1988 to 2008 Week 48	OVID	Nov 28, 2008	334
Health and Psychosocial Instruments 1985 to Oct 2008	OVID	Nov 28, 2008	110
AARP Ageline 1978 to Oct 2008	OVID	Nov 28, 2008	324
CINAHL Plus with Full Text 1937 to present	EBSCOHost	Dec 1, 2008	433
ProQuest Dissertations and Theses; and ABI/INFORM Global 1971 to Current	ProQuest	Dec 1, 2008	363
PsycINFO 1806 to Nov Week 4 2008	OVID	Dec 1, 2008	153
Web of Science: SCI-Expanded, SSCI, A&HCI, 1900-2008	ISI Web of Knowledge	Dec 1, 2008	502
The Cochrane Library	The Cochrane Library	Dec 2, 2008	3

Databases searched

Search terms: (minimum data set$ OR resident assessment instrument$ OR rai OR interrai OR mds).mp. AND (long-term care OR nursing home$ OR care home$ OR continuing care OR facility living).mp. AND (exp quality of health care/OR (quality OR outcome$ OR performance).mp.) OR (Reproducibility of Results/OR (valid$ or accura$ or reliab$).mp.)

*Note*: The "mp" field qualifier in OVID MEDLINE searches the following fields: title, original title, abstract, name of substance word, and subject heading word. In other databases it searches similar text fields. The "sh" field qualifier indicates a subject heading search. "Exp" indicates an "exploded" subject heading. The terminal qualifier "/" also indicates a subject heading. "*" and "$" are truncation symbols.

All searches were limited to English language materials where possible.

## Inclusion criteria

The searches were limited to literature in the English language and to articles or reports of research published up to December 2008. Included publications reported research with a clearly stated purpose, the primary intent of which was to examine an aspect of validity and/or reliability of the RAI-MDS 2.0 QIs. We excluded publications that discussed aspects of validity or reliability of the RAI-MDS 2.0 QIs if that was not the original or primary purpose of the study.

## Screening

**Figure 1:**
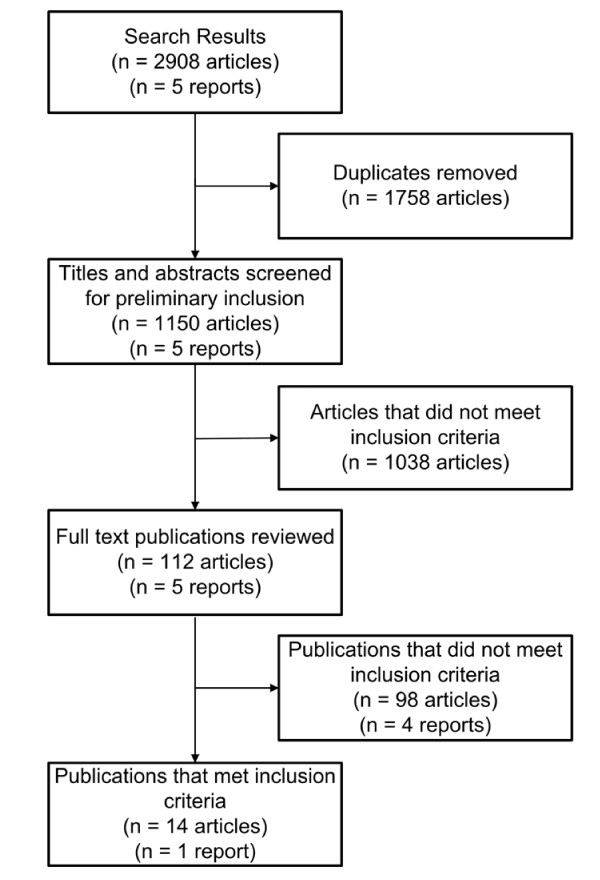
**Screening process for relevant studies**.

Following de-duplication all references were individually scrutinized by AMH to assess their potential relevance. This approach identified 112 articles, which were retrieved in full text. A detailed description of the search screening process is outlined in Figure 1. Four potentially relevant reports were also identified from the website search and one additional report resulted from the Google Scholar search. A total of fourteen articles and one report (representing fourteen studies) met the inclusion criteria.

## Data extraction

Data from the final set of included articles and reports examining the validity and reliability of individual indicators (n = 15) were extracted by AMH and are reported in Additional File 1. A second member of the research team (JES) checked the extracted data for accuracy.

## Quality assessment

An assessment of the methodological quality of the included articles and the report was undertaken by JES using an instrument designed for critical appraisal of observational studies [29]. In accordance with the recommendations of Sanderson et al. [30], we selected this instrument because it covers a small number of key domains, it is specifically designed for assessment of any observational study, it comprises a simple checklist rather than a scale, and there is evidence that it was developed using a range of literature sources. The instrument addresses four broad domains, each addressing several sub domains as follows: (1) What the study is about? (relevance of the study to the needs of the project, and does the paper address a clearly focused issue in terms of the population studied, outcomes considered, and the aims stated?); (2) Do I trust it? (appropriateness of methods used, appropriateness of the population studied, confounding and bias, and follow-up); (3) What did they find? (tables/graphs labeled and understandable, correct statistical methods, conclusions supported by information cited); and (4) Are the results relevant locally? (results applicable to local situation, all important results considered, cost-information provided). We adapted the instrument for our purposes by removing items particular to case control studies, since this design was not used for any of the included studies. Because all relevant studies were included in the review, we omitted the concluding question that asks whether the study is accepted for further use. Further, we added a ‘not applicable' option to the response categories. Items within each sub domain were then categorized as follows: yes (criteria met), no (criteria not met), cannot tell, or not applicable.

## Results

### Description of the studies

The included studies fell into two broad categories, those that examined individual quality indicators and those that examined multiple indicators. One report and one article [10,31] present findings arising from the single study that examined multiple indicators. Thirteen articles present findings from studies examining single QIs [32-44]. A total of ten QIs were examined individually (Additional File 1).

All studies were conducted in the United States. They ranged in size to be inclusive of one or multiple LTC facilities (maximum of 209 facilities), while the number of residents included ranged from n = 109 to n = 5758. Studies used one of two approaches: 1) Comparison between RAI-MDS 2.0 data routinely collected by facility staff and that collected by trained research nurses (n = 2), or 2) Comparison between data collected using the RAI-MDS 2.0 instrument and that collected using another method (for example, another instrument, chart documentation, direct observation, or interview) designed to measure the same resident characteristics (n = 12). The study of multiple indicators adopted the former approach. With one exception [43], the studies examining single indicators used the latter approach. Of the single indicator studies, eight compared data collected in the highest quartile facilities (i.e., for prevalence of the indicator) with that of the lowest quartile facilities. Studies that examined individual QIs have tended to be limited by a number of factors including sample size, low facility consent rates, variability in recruitment rates for study groups, and generalizability (all studies were conducted in the U.S.).

Methodological quality of the 14 studies was assessed according to four domains, as presented in Additional File 2. There was considerable consistency between studies with respect to whether they met the criteria specified within each domain. The majority of the studies met the quality criteria in domains 1 (What is the paper's focus?) and 3 (What did they find?). With respect to quality assessment, the least favorably ranked sub domain was that of *confounding and bias *(within Domain 2 - Do I trust it?); all 14 studies scored either negatively or did not provide sufficient information for assessment of one or more of the items in this sub domain. A second area of poor performance was the sub domain of *providing cost information *(within Domain 4 - Are the results relevant locally?); none of the included studies provided cost information. It was not the purpose of any of the studies, however, to conduct an assessment of the cost implications of undertaking the RAI-MDS 2.0 assessments. In the following, the results are presented, first, for the large study conducted in 2003 that examined multiple indicators. This is followed by results from studies conducted to examine individual indicators. These studies are grouped according to the respective indicators.

### Study examining validity and reliability of multiple RAI-MDS 2.0 quality indicators

In 2003, Abt Associates, contracted by the CMS, undertook a large study of data derived from RAI-MDS 2.0 with a goal of validating and testing the inter-rater reliability of 38 chronic care QIs, many of which were already in use by the CMS. One article and one report present validity and reliability findings of this study [10,31]. The study sample comprised 209 free-standing and hospital-based facilities in six U.S. states. Within each facility the researchers attempted to sample 30 residents, resulting in the inclusion of almost 6000 residents. Trained research nurses, who had demonstrated high inter-rater reliability, independently conducted observational assessment of the residents, undertook chart reviews and interviewed staff about resident behavior. The research nurses' ratings were used as the 'gold standard' and were compared with routinely collected RAI-MDS 2.0 data. Reliability was assessed using kappa statistics and percentage agreement to compare independent ratings conducted by trained research nurses with those of facility nurses for individual data elements and a subset of the QIs. In their final report of the national validation study, Morris et al. [31] concluded that strong evidence existed in support of several of the QIs' capacity to reliably measure relevant dimensions of facility performance; kappa coefficients for all QIs, with one exception (percentage of *residents engaging in little or no activity*) were greater than .4 (which the researchers interpreted as indicative of acceptable inter-rater reliability).

To validate the meaningfulness of the QIs the researchers [31] examined the strength of the relationship between the QIs and measures of practices, processes structures and outcomes, which, in theory, were predictors of high performance on specific QIs. Predictors were identified by asking multidisciplinary expert panels to recommend criteria against which to validate the QIs and to formulate hypotheses about factors distinguishing "good" from "poor" performance. For each QI, a combination of observational, survey and medical record review data were collected to measure hypothesized predictors of good performance. All data were reviewed and individual items or combinations of items were recommended for use in discriminating between "good" and "poor" performance. These validation elements were then classified as preventive strategies (actions designed to prevent quality problems), or responsive strategies (actions initiated in response to the identification of quality problems). The chronic care QIs (n = 12) found to have the highest level of validity were recommended for use in public reporting (Table 3). However, not all were included in the publically reported data on the CMS Nursing Home Compare website. Validity was based on the QIs' strong association with quality of care activities, including preventive and responsive care strategies, as elicited from medical record, survey and observational data.

**Table 3:** Quality Indicators, derived from RAI-MDS 2.0, tested by Abt Associates Inc.

	Indicator
	Percent of residents with inappropriate behavior (high risk and low risk)**
**Prevalence**	Percent of residents with inappropriate behavior (high risk)
	Percent of residents with inappropriate behavior (low risk)
	Percent of residents engaging in little or no activity
	Percent of residents with indwelling catheters*
	Percent of residents who are bladder or bowel incontinent (high and low risk; high risk; low risk)*
	Percent of residents with a urinary tract infection*
	Percent of residents who have fallen
	Percent of residents with infections*
	Percent of residents with a feeding tube
	Percent of residents with a low BMI
	Percent of residents who have unexplained weight loss**
	Percent of residents with pain*
	Percent of residents with pressure sores (high and low risk)*
	Percent of residents with pressure sores (high risk)*
	Percent of residents with pressure sores (low risk)
	Percent of residents with burns, skin tears or cuts
	Percent of residents in physical restraints
	Percent of residents on antipsychotics without a diagnosis of psychosis (high risk and low risk)**
	Percent of residents on antipsychotics without a diagnosis of psychosis (high risk)
	Percent of residents on antipsychotics without a diagnosis of psychosis (low risk)

**Incidence**	Percent of residents who had an unexpected loss of function in some basic daily activities*
	Percent of residents with worsening function in some basic daily activities*
	Percent of residents who have improved in their ability to function
	Percent of residents who have declined in their ability to locomote*
	Percent of residents who walk as well or better than the previous assessment*
	Percent of residents whose cognitive ability has worsened
	Percent of residents whose ability to communicate has worsened
	Percent of residents with symptoms of delirium
	Percent of residents whose behavior has worsened**
	Percent of residents who have become more depressed or anxious
	Percent of residents with a new indwelling catheter
	Percent of residents with worsening bowel continence
	Percent of residents with worsening bladder continence*
	Percent of residents with worsening pain
	Percent of residents with worsening pressure sores**

* Found to have the highest level of validity and highly recommended by Abt Associates for use by CMS and nursing homes

** Rejection of these quality indicators was recommended by Abt Associates at the time of the research

Also reporting on the national validation study data to measure inter-rater reliability of the RAI-MDS QIs, Mor, et al., [10] presented kappa statistics calculated for 100 RAI-MDS data elements and 22 QIs derived from these data elements. These were calculated at the facility level as well as at the level of individual residents. Average kappa scores across all facilities were calculated for the 22 QIs. These ranged from .23 (which the researchers interpreted as unacceptable agreement) to .87 (which the researchers interpreted as excellent agreement). Levels of agreement were reported for select QIs. For two QIs, *infection *and *little or no activity*, agreement was described as "barely adequate" (Results, ¶ 6), with kappa scores of .39 and .23, respectively. Four QIs (*prevalence of incontinence, prevalence of tube feeding, prevalence of low body mass index*, and *prevalence of antipsychotic use) *had very good agreement [8], with kappa values exceeding .8. While, on average, a reasonable level of agreement in the QI ratings was achieved, there was wide variation between facilities in the kappa values for the QIs. For some QIs, almost half the facilities failed to achieve adequate reliability. While 18% of facilities had good to very good agreement [8] (kappa >.75) on 12 or more QIs, 16% of facilities had poor to fair agreement [8] (kappa <.4) on over six of the QIs. The researchers concluded that the inter-rater reliability for most QIs ranged from "adequate to good" [[10] Discussion, ¶ 1]. The between-facility variation revealed that most facilities had reasonable reliability for most QIs, while some facilities had unacceptably low kappa scores for several QIs.

### Studies examining validity and reliability of single quality indicators

Studies that examined validity of single QIs aimed to determine whether the QIs reflected care processes associated with the aspect of care being measured, and/or validation of QIs with instruments measuring the same construct and known to be valid and reliable. A limited number of QIs have been studied using one of these approaches. An outline of the findings follows.

#### Falls

In order to evaluate the validity and reliability of falls reporting using the RAI-MDS 2.0, Hill-Westmoreland & Gruber-Baldini [32] examined the level of agreement between falls recorded by facility staff in the RAI-MDS 1.0/2.0 and falls recorded in medical charts. Data were collected for two RAI-MDS items, *fell in the past one to thirty days *and *fell in the past thirty one to one hundred and eighty days*. Nurses trained in data abstraction collected falls events data from the medical charts for the same time intervals. They found a 65% agreement rate for a 30-day timeframe, with a resulting statistically significant kappa score of .29, indicating fair agreement [8]. For a 180-day timeframe they found agreement in 75% of cases, with a statistically significant kappa score of .5, indicating moderate agreement [8]. The researchers recommended use of a 180-day interval in the future to reduce measurement error. Medical chart data revealed that 49% of the sample experienced a fall, while according to the RAI-MDS data 28% had fallen during the 180-day interval. The researchers concluded that the RAI-MDS underreported falls and recommended caution in use of the RAI-MDS data as the only indicator of falls. Lack of a clear definition for a fall was hypothesized as one possible reason for the variation seen in reporting between individuals and facilities.

#### Depression

Three studies [33-35] have been undertaken specifically to validate the RAI-MDS *depression *QI. In 2001 Schnelle et al. [35] measured the sensitivity (proportion of residents correctly identified as depressed out of all residents experiencing depression) of the RAI-MDS *depression *QI in two LTC facilities. One facility ranked as having a low and another as having a high prevalence rate on the *depression *QI were included. The researchers measured residents' symptoms of depression in an interview and compared the results with documented measures for mood in the most recent RAI-MDS. The researchers found that the proportion of residents they assessed as having probable depression was not significantly different between the two facilities. The researchers argued that the ability to detect depression accounted for the difference between the two facilities and that the higher prevalence site should not be considered to have a greater problem with depression in comparison with the lower prevalence site. They contended that the lower prevalence site required an intervention to improve the detection of depressive symptoms. Schnelle et al. concluded that their results suggested the *depression *QI measured the ability of staff to detect depressive symptoms rather than the actual prevalence rate of depression.

Simmons et al. [34] tested the validity of RAI-MDS QI data by comparing it with the prevalence of depressive symptoms determined through independent assessments by researchers. Further, they examined whether LTC facilities that scored differently for the RAI-MDS *depression *QI provide different depression-related care. Residents (n = 396) in facilities rated in the highest (n = 4) and lowest (n = 10) quartiles for the *depression *QI were studied. The researchers employed direct observation, resident interviews and medical chart review over three consecutive 12-hour days. The prevalence of independently assessed depressive symptoms was significantly greater than that reflected in the RAI-MDS QI for facilities in the highest and lowest quartiles. Furthermore, the prevalence of depressive symptoms in the highest and lowest quartile facility groups was similar. While documentation of depressive symptoms was significantly higher in facilities in the highest quartile, this was not correspondingly associated with implementation of appropriate care processes. The results of this study led the researchers to "strongly suggest that the current MDS depression quality indicator should not be interpreted as discriminating either differential rates of depression or care quality in relation to depression" [[34] p. 563].

Heiser [33] tested the validity of the RAI-MDS *depression *QI in one LTC facility by comparing rates of depression identified using the RAI-MDS depression scale with those identified using two instruments that are known to be valid: the Geriatric Depression Scale (GDS) Short Form and the Schedule for Affective Disorders and Schizophrenia (SADS). Trained research staff administered the GDS and the SADS. Their findings cast doubt over the validity of the RAI-MDS *depression *QI because the QI correlated poorly with the valid instruments (indicating a lack of convergent validity) and exhibited inferior sensitivity and specificity. The GDS detected more residents with depression than did the RAI-MDS *depression *QI - 35% versus 3%, respectively. The GDS identified residents with depression as accurately as the SADS (at statistically significant levels), but the RAI-MDS *depression *QI had a significantly lower agreement rate. The researchers concluded that the RAI-MDS is not the most accurate measure of depression in long term care facilities.

#### Depression without treatment

Zisselman et al. [36] evaluated the validity of the RAI-MDS *depression without treatment *QI using a retrospective chart review of psychotropic medications, psychiatric diagnosis, mental health evaluation and treatment for all residents (n = 538) in one LTC facility. Of the residents who were recorded as depressed and not receiving treatment, approximately half were actually receiving appropriate treatment. The researchers warned their results suggested "the presence of the quality indicator, *depression without treatment*, may not accurately capture clinically depressed ... residents in need of mental health intervention" [[36] p. 41].

#### Incontinence

To assess the validity of the RAI-MDS incontinence QIs, Schnelle et al. [37] compared care processes in LTC facilities rated in the highest (n = 7) and lowest (n = 7) quartiles for the RAI-MDS incontinence QI, *prevalence of incontinence; *and facilities rated in the highest (n = 9) and lowest (n = 7) quartiles for the RAI-MDS incontinence QI, *prevalence of incontinence without a toileting plan*. The researchers observed the implementation of 9 care processes for 12-hours per day over 3 days. They also interviewed residents, evaluated residents' physical performance and reviewed documentation. The results indicated that facilities with lower rates on both of the incontinence QIs had statistically significantly higher documentation for evaluation of incontinence history and for toileting assistance by staff. Interviews with competent residents, however, indicated no difference in the level of toileting assistance provided by staff in the two groups of facilities. In addition, the researchers found no difference in frequency of scheduled toileting assistance for incontinent residents who were rated as receiving such assistance compared with residents who were recorded as *not *receiving scheduled toileting assistance. The researchers concluded, "the MDS incontinence quality indicators were not associated with clinically important differences in related care processes" [[37] pp. 909-910].

#### Urinary tract infection

To establish the validity of the RAI-MDS in identifying cases of urinary tract infection, Stevenson et al. [38] compared the RAI-MDS data for urinary tract infection (UTI), with data arising from active prospective surveillance in LTC facilities (n = 16). The researchers concluded that "when used to detect residents with UTIs ... [the RAI-MDS] appears to greatly overestimate the number of cases while adequately screening out residents without UTIs" [[38] p. 708]. Of the RAI-MDS data entries that indicated a resident had experienced a UTI within the past 30 days, only 13.9% could be validated as correct through active surveillance or medical chart review. On the other hand, 98.2% of entries that indicated the resident had not experienced a UTI within the last 30 days could be validated as correct. The researchers suggest that provision to assessors of more explicit definitions of UTIs may help to overcome the problem of false positive reports. In January 2008, clarification of the term "symptomatic", with respect to a urinary tract infection, was made in a revision of the CMS RAI Version 2.0 Manual.

#### Weight loss

Some evidence for validity of the RAI-MDS *weight loss *QI was provided by Simmons et al. [39] who studied LTC facilities in the highest (n = 5) and lowest (n = 11) quartiles to determine whether prevalence of the RAI-MDS *weight loss *QI was consistent with weight loss related care processes. Over three consecutive 12-hour days the researchers used direct observation during meal times, interviews of residents, and analysis of medical chart documentation to examine care processes related to weight loss. Weight loss was significantly greater in residents in the highest quartile group according to RAI-MDS data and monthly weight recorded in the medical records. Further, the highest quartile group had a greater proportion of residents with weight loss risk factors. With respect to care processes, the researchers reported that staff in the lowest quartile group of facilities consistently offered verbal prompts and social interaction to a larger proportion of residents at meal times. Simmons et al. concluded that the RAI-MDS weight loss QI is able to discriminate differences in prevalence of weight loss between facilities, suggesting concurrent validity of the QI.

#### Bedfast

Contributing evidence for the validity of the RAI-MDS *bedfast *QI, Bates-Jensen and colleagues [40] compared LTC facilities that scored in the highest (n = 7) and lowest quartile (n = 8) for the *bedfast *QI. The researchers interviewed residents (n = 451) and conducted direct observation. The observations entailed hourly checks for one day from 0700 to 1900. The proportion of time residents in the higher prevalence group were observed in bed was significantly higher than that observed in the lower prevalence group. Furthermore, the residents in the higher prevalence group were observed to experience more activity and reported receiving more assistance with mobility than did residents in the lowest quartile. The researchers reported that RAI-MDS scores in all facilities underestimated the number of bedfast residents. While the bedfast QI discriminated according to facilities in which residents spent greater time confined to bed, the researchers concluded that it failed to identify differences in activity and assistance with mobility. Bates-Jensen et al. [40] found facilities with higher bedfast prevalence provided a higher level of activity and mobility assistance.

#### Restraint

Validity evidence in relation to the RAI-MDS *prevalence of restraint *QI was provided by Schnelle et al. [41] who examined whether the QI reflected differences in care. They studied facilities that rated in the highest (n = 6) and lowest (n = 8) quartiles on the RAI-MDS *prevalence of restraint *QI, a measure of use of restraining devices when residents are *out of bed*. Researchers directly observed the use of restraining devices over 12-hours per day for three days. In the facilities with higher restraint use, residents spent more time *in bed *during the day, had bed rails in place more often, and received less assistance with eating. On the other hand, there were no observed differences between the highest and lowest restraint-use facilities when it came to use of restraints when residents were out of bed, care processes in restraint management, gait or balance issues, or activity levels. Schnelle et al. concluded that although the differences between the groups did not reflect a difference in the use of restraining devices when the resident was **out of bed **(which is what the prevalence of *restraint *QI is designed to measure); differences were detected in other important aspects of the quality of care.

#### Pressure ulcers

Bates-Jensen et al. [42] studied residents (n = 329) in LTC facilities to test the assumption that facilities with lower RAI-MDS *pressure ulcer *(PU) QI scores provide better pressure ulcer care, thereby providing evidence relating to validity of the *pressure ulcer *QI. The researchers examined whether facilities that scored in the highest quartile (n = 10) differed from facilities in the lowest quartile (n = 6) in the PU care provided. Process indicators were measured from medical record data, direct observation and the use of wireless thigh movement monitors. According to the findings of this study there were no differences between the two groups for most PU care processes. However, the facilities with higher PU prevalence rates did use pressure-reducing surfaces more frequently and were more effective in documenting the location, size, stage and existence of necrotic tissue when a PU was present. Despite documenting 2-hour repositioning in the medical record for almost all residents, 2-hourly repositioning was not routinely conducted, according to the observational data, in either group of LTC facilities. Bates-Jensen et al. concluded that the MDS PU indicator was not an effective measure of the quality of PU care in LTC facilities. Further, they warned that unless information about the meaning of the indicator was provided with the results, the PU QI scores could be misleading [[42] p. 1203].

#### Pain

Although the *pain *QI was not included in the publically reported data on the CMS Nursing Home Compare website, it was developed as a measure of quality of care [31]. Wu, Miller, Lapane, Roy & Mor [43] assessed the validity of RAI-MDS pain reporting, comparing "gold standard" research nurses' pain ratings for almost 3,500 non-hospice residents with those of staff working in low, medium or high hospice-use LTC facilities. In examining the frequency of false positive and false negative errors in ratings of severe pain, the researchers found that staff of medium hospice-use facilities were less likely to make such errors in their RAI-MDS documentation. In addition, the facility characteristics and location (by state) explained over 50% of the variance in reporting. The researchers concluded that the characteristics of the facility are systematically associated with pain rating scores and may bias comparisons for the *pain *QI.

Cadogan et al. [44] examined the validity of the *pain *QI in reflecting pain-related care processes. They compared these processes for facilities that scored in the highest (n = 8) and lowest quartile (n = 8) for the *pain *QI. The researchers evaluated the pain-related care processes using resident interviews and medical record documentation review (n = 255). The interviews revealed a significantly higher proportion of residents reported symptoms associated with chronic pain in the highest quartile facilities. In contrast with the pain prevalence indicator, the interviews also revealed a significantly higher prevalence of pain in residents in the lowest quartile group. Furthermore, for residents in the highest quartile group, documentary analysis showed a statistically significantly higher proportion received pain assessments by nurses and doctors, pain medications, and documentation of their response to treatment in comparison with those in the lowest quartile. While the researchers concluded that the RAI-MDS *pain *QI accurately differentiates the prevalence of pain between facilities (concurrent validity), they recommended caution when interpreting the results. Specifically, they noted that high pain prevalence scores were associated with more frequent pain assessment and appropriate pain-related care practices, as opposed to poor care quality [[44] p. 281].

## Discussion

### Are the indicators valid and reliable?

Our review suggests that the evidence for the validity and reliability of the RAI-MDS QIs is mixed. While one study demonstrated good reliability and validity of certain QIs, it was conducted under research conditions. Some studies conducted in "real world" conditions have revealed the potential for systematically biased data with under-reporting of some QIs, such as the pain, falls and depression QIs, and over-reporting of others, such as the prevalence of UTIs.

Considerable research has been undertaken to validate RAI-MDS versions 1.0 and 2.0 data elements [14-17]. In addition, the reliability of the data elements in these versions has been tested comprehensively [9,12,19]. Mor [6], however, argued that although extensive research has lent support to the construct and predictive validity of the data elements within the RAI-MDS, little research exists to confirm the validity of the QIs, with respect to their consistency with other measures of performance and in regards to their ability to accurately reflect the effects of change in practices associated with high quality care.

To determine whether any publications on the subject of the reliability or validity of the RAI-MDS 2.0 quality indicators had been published since our original search, we executed a new search on July 6, 2009, and located one new article that is relevant to this review [45]. Using data collected using RAI-MDS 2.0 during 2001 and 2002 for the U.S. national validation study [31], discussed previously, the researchers investigated associations between measurement bias and characteristics of facilities and residents. Data from 5344 paired MDS assessments that had been independently conducted in 206 nursing homes by facility staff and research nurses were analyzed [45]. Analysis involved multivariate, multi-level modeling of 29 RAI-MDS 2.0 items, of which many are included in the derivation of the QIs. The researchers found that resident characteristics accounted for little or no variation in coding. However, facility characteristics accounted for 4-20% of coding differences, and facility location (based on state) explained 13-34% of variation in data quality. The researchers expressed concern that the magnitude of the measurement bias observed may threaten the validity of the QIs [45].

### Use of the RAI-MDS quality indicators to inform quality improvement programs

Karon and Zimmerman [3] stress that the QIs are indicators of potential issues and are not measures of quality. Hence, the indicators should be used as an initial step in the process of evaluating the quality of care. Karon and Zimmerman state, "the final decision of whether or not there is a quality problem, and the nature of that problem, requires careful and skilled investigation by clinical experts" [[3] p. 254].

While the RAI-MDS 2.0 QIs provide a useful starting point for further evaluation and analysis of identified quality issues, caution should be exercised when interpreting the QI results. The results of this review suggest that further work will be required before they are established as valid assessments. The evidence of systematic bias and the degree of variation in the indicators related to facility characteristics versus variations in quality or resident characteristics suggests that much more attention needs to be paid to the quality and accuracy of RAI-MDS data capture in long term care facilities. Investment in resources to support staff to undertake assessments and to utilize the data in care planning and evaluation is important in promoting accuracy of the data and to ensuring that the data is valued. The indicators should be considered in the context of other evidence relevant to care quality and explanations for the apparent existence of poor quality, according to the indicator, should be sought and carefully explored. High QI scores (indicating poor care quality) may actually reflect well-developed skills of staff in identifying a clinical condition and may be associated with the use of appropriate care processes [35,44].

Facility administrators and direct care providers can use QIs, in the context of other evidence, to identify potential quality issues, analyze the extent and impact of quality issues, inform the development of quality improvement initiatives, track response to quality initiatives, benchmark their facility's performance with regional, provincial and national averages, and provide a method for monitoring the accuracy of RAI-MDS documentation [1,46,47]. A recent study suggested that QI reports play a central role in quality improvement initiatives, enabling identification and tracking of quality problems, providing a benchmark with which to compare the facility's quality of care, and providing a method for monitoring the accuracy of RAI-MDS documentation [46].

This review has some limitations. First, we included English language publications only. Second, although a comprehensive search was constructed and executed with the assistance of a health sciences librarian, it is possible that relevant publications were not identified. The nature of search engines and bibliographic databases means that replication of the search for the same time frame and using the same search criteria will almost certainly fail to produce identical results [48]. Third, despite using a systematic procedure, the subjective nature of the screening process, data extraction and quality assessment may have influenced the findings [48]. Finally, heterogeneity between studies with respect to design and the quality indicators reviewed enabled descriptive analysis only.

## Conclusion

To summarize, the findings presented in this review indicate that the strength of the evidence with respect to the reliability and validity of the RAI-MDS 2.0 QIs is limited, and further research in this area is warranted [2,6]. While the QIs provide a useful tool for quality monitoring and with which to inform quality improvement programs, caution should be exercised when interpreting the QI results. Importantly, the results should be contextualized and interpreted in conjunction with other valid and reliable sources of information and evidence about care processes. Finally, this review indicates the need for further validation of the RAI-MDS 2.0 QIs.

## Competing interests

The authors declare that they have no competing interests.

## Authors' contributions

AMH designed the review, developed the search strategy, conducted the selection, undertook the data extraction, checked the quality assessment and wrote the manuscript. DM, SM and CJ developed the research question and provided critical commentary on initial versions of the manuscript. JES checked the data extraction, conducted the quality assessment and provided critical commentary on the final manuscript. GT provided critical commentary on the final manuscript. CE provided valuable advice during the development and conduct of the study and provided critical commentary on the final manuscript. All authors have read and approved the final submitted manuscript.

## Pre-publication history

The pre-publication history for this paper can be accessed here:

http://www.biomedcentral.com/1472-6963/10/166/prepub

## Supplementary Material

Additional file 1 **Studies examining validity and reliability of RAI-MDS quality indicators**. Data extracted from included studies Click here for file

Additional file 2 **Quality assessment of included studies**. Quality assessment data from included studies Click here for file
