# Supplementary material for: The Resident Assessment Instrument-Minimum Data Set 2.0 quality indicators: a systematic review
Source: BMC Health Serv Res. 2010 Jun 16;10:166. doi: 10.1186/1472-6963-10-166 (PMC2914032; doi:10.1186/1472-6963-10-166)
Supplement: Additional file 1 — Studies examining validity and reliability of RAI-MDS quality indicators. Data extracted from included studies [file 1472-6963-10-166-S1.DOC]

**Additional File 1 - Studies examining validity and reliability of RAI-MDS quality indicators**

| ***QI evaluated*** | ***Source*** | ***Setting and sample*** | ***Design*** | ***Data collection*** | ***Key results*** |
| --- | --- | --- | --- | --- | --- |
| Multiple QIs | Morris et al., (2003); Mor, Angelelli et al. (2003) | 6 states, United States  209 LTC facilities, stratified sample  Up to 30 residents, recently assessed using RAI-MDS, per facility were sampled  n=5758 | - Cross-sectional study - Morris et al. examined the validity and reliability of 38 QIs applicable to LTC - Mor, Angelelli et al. examined reliability of QIs, comparing poor and well-performing facilities across 22 QIs | - Expert panels developed hypotheses regarding the structures and processes to promote quality in relation to specific QIs - Observational, survey and record review data measuring structures and processes related to the QIs were collected - Trained research nurses conducted resident assessments, collecting approximately 100 data elements - Comparisons were made between the research nurses’ assessments and those of the facility staff, as recorded in the RAI-MDS | Morris et al., (2003), in the final Abt Associates Inc. report, reported evaluation of 38 QIs applicable to LTC (chronic) populations. They highly recommended 14 LTC QIs for use, based on the strength of the evidence for their validity. Another 17 QIs were reported as appropriate, though not highly recommended, for use as indicators of care quality. The researchers recommended against the use of 5 QIs, based on limited evidence for their validity. Average weighted kappa levels ranged from .28 to .89. The QI, *percentage of residents engaging in little or no activity,* was the only QI considered to perform poorly (kappa <.4) with a kappa level of .28.  Mor, Angelelli et al. (2003), in a peer-reviewed publication arising from this study, report that kappa levels for 20 out of 22 QIs were “adequate” (i.e ≥.4). The researchers report actual kappa values for 12 QIs. These range from .23 to .87. The average kappa level was greater than .80 for 4 QIs. Facility characteristics were not associated with reliability of the QIs. |
| Falls | Hill-Westmoreland & Gruber-Baldini (2005) | Maryland, United States  56 LTC facilities  n=462 residents, randomly sampled | - Secondary analysis of longitudinal data - MDS data compared with that extracted from chart documentation | - Data extracted from charts - RAI-MDS data | According to chart documentation, 49% of the sample experienced a fall. However, MDS data revealed that only 28% of the sample had fallen. The researchers concluded that the MDS data underreported falls. |
| Depression | Heiser (2004) | United States  1 LTC facility  n=348 residents | - Cohort study - Compared depression identification rates and validity of the MDS with the GDS short form | - Routinely collected MDS data for the depression QI were retrieved - Residents were assessed for depression using the GDS short form - Residents were also assessed with the Schedule for Affective Disorders and Schizophrenia (SADS) as the standard | The GDS detected more residents with depression than did the MDS QI– 35% versus 3%, respectively. The GDS identified residents with depression statistically significantly as accurately as SADS, but the MDS QI depression results were significantly lower than the GDS. Sensitivity of the MDS QI (proportion of residents determined to be depressed out of all the residents who were experiencing depression) when compared with the SADS was .88, while the corresponding sensitivity of the GDS was .91. |
|  | Simmons et al. (2004) | California, United States  14 LTC facilities  n=396 residents | - Cross sectional - Compared LTC facilities in the upper and lower quartile for the QI | - Medical record analysis - Direct observation - Interviews (using the GDS short form) with residents - RAI-MDS data | The depression QI underestimated the prevalence of depressive symptoms in LTC facilities across the board, but particularly in facilities reporting very low depression rates. Documentation of depression was not associated with better care processes. A greater proportion of individuals with GDS scores of > 5 (probable depression) also displayed behavioral symptoms, when compared with individuals with GDS scores ≤5 (p<.001) |
|  | Schnelle et al. (2001) | California, United States  2 LTC facilities  n=91 residents | - Cohort study - Compared LTC facilities in the upper and lower quartile for the QI | - Researchers measured residents’ depression symptoms using the GDS short form and compared their results with those documented on the RAI-MDS by staff. | The findings of this study suggest that the skills of staff to detect depression may influence the reported prevalence of depression. The percentage of residents found to have probable depression were similar across groups using the GDS. However, there was a significant difference (p<.01) between sites on QI estimates of the prevalence of depression. |
| Depression without treatment | Zisselman et al. (2002) | Pennsylvania, United States  1 LTC facility  n=538 residents | - Cohort study - Retrospective chart review of residents identified having depression without treatment | - Medical record analysis - RAI-MDS data | Of residents recorded as depressed and not receiving treatment, about half were actually receiving appropriate treatment (medication followed by geropsychiatrist consultation). Results suggest that the *depression without treatment* QI may not detect clinically depressed residents. |
| Urinary incontinence | Schnelle et al. (2003) | California, United States  14 LTC facilities  n=347 residents | - Cross-sectional study - Compared LTC facilities in the upper and lower quartile for the QI | - Medical record analysis - Direct observation - Interviews with residents - Physical performance evaluation - RAI-MDS data | The urinary incontinence QI did not reflect differences in clinical care processes. There was a discrepancy between documentation of care and resident reports of care provided. The results “strongly suggest that the MDS incontinence quality indicators do not reflect differences in the quality of incontinence care between homes” (p. 920). |
| Urinary tract infections | Stevenson et al. (2004) | Idaho, United States  16 LTC facilities  n=6947 RAI-MDS entries to answer the question “UTI in last 30 days”, of theses n=478 cases could be matched to surveillance data | - Prospective surveillance | - RAI-MDS entries for a 9-month period were compared with prospective surveillance undertaken by the facility using standardized data collection forms. - Information was abstracted for the medical record for evidence of infection e.g. fever, culture results, antibiotics prescribed | Of residents documented as having a UTI on the MDS, only 13.9% could be validated (‘yes’ entries had low predictive value for identifying UTIs). Thus, when used to measure the prevalence of urinary tract infections the MDS appeared to overestimate the prevalence. On the other hand, 98.2% of residents determined not to have a UTI did not in fact have a UTI (‘no’ entries were highly predictive of dismissing UTIs). |
| Weight loss | Simmons et al. (2003) | California, United States  16 LTC facilities  n=513/1497 (34%) residents consented to participate  n=464/1497 (31%) completed the study | - Cross-sectional study - Compared LTC facilities in the upper and lower quartile for the QI | - Medical record analysis - Direct observation - Interviews with residents - MDS data | Facilities with lower weight loss prevalence had fewer at risk residents and staff provided significantly more verbal prompts and social interaction during meals. The weight loss QI in this sample differentiated difference in the prevalence of weight loss between facilities. |
| Bedfast | Bates-Jensen et al. (2004) | California,  United States  15 LTC facilities  n=451 residents | - Cohort study - Compared LTC facilities in the upper and lower quartile for the QI | - Medical record analysis - Direct observation - Interviews with residents | The bedfast QI discriminated between facilities in the upper and lower quartiles for number of bedfast residents and length of time spent in bed.  Mean length of time in bed  Lower quartile: 17 hours/24 hours (SD 3.3)  Upper quartile: 18 hours/24 hours (SD 3.5)  (t=2.852, p=.005)  Number of bedfast residents  Lower quartile: 17/207 (8%) in bed for 80% of observation period  Upper quartile: 42/232 (18%) in bed for 80% of the observation period  (χ^2^=9.199, p=.002)  However, activity and mobility care processes were not reflected in the QI scores. Upper quartile facilities offered more activity and mobility care (65% compared with 33%; χ^2^=11.548, p=.001). All facilities underestimated the number of bedfast residents. |
| Restraint | Schnelle et al. (2004) | California, United States  14 LTC facilities  n=413 residents | - Cross-sectional study - Compared LTC facilities in the upper and lower quartile for the QI | - Medical record analysis - Direct observation - Interviews with residents - Wireless thigh movement monitors | The LTC facilities restraint QI scores did not reflect differences in out-of-bed use of restraints, physical activity or care process measures. But, for in-bed restraint use the score did differentiate between facilities (73% in low prevalence homes and 81% in high prevalence homes; χ^2^=3.7, p<.05) – despite the fact that the restraint QI is not designed to measure these differences. |
| Pressure ulcer | Bates-Jensen et al. (2003) | California, United States  16 LTC facilities  n=329 residents | - Descriptive, cohort study - Compared LTC facilities in the upper and lower quartile for the QI | - Medical record analysis - Direct observation - Interviews with residents - Wireless thigh movement monitors | The PU QI accurately differentiated between LTC facilities in the upper and lower quartiles but failed to reflect difference in most PU care processes. In fact, facilities that were high on the PU indicator used pressure-reducing surfaces more often and had better documentation of wound characteristics when PUs were present. Neither group repositioned residents 2-hourly even though 2-hourly repositioning was documented in the medical record for almost all residents. |
| Pain | Wu et al. (2005) | California, Illinois, Missouri, Ohio, Pennsylvania and Tennessee, United States  178 LTC facilities  n=3469 non-hospice residents | - Compared staff adherence to pain assessment protocols in hospice LTC facilities with non-hospice facilities - Used data from the national validation study | - Researchers measured residents’ pain frequency and intensity and compared their results with those documented on the MDS by staff. | The characteristics of the facility were systematically associated with over- or under-rating of pain and may bias comparisons on this QI. The quality of pain documentation for non-hospice residents was better in facilities with medium hospice use. The pain variables – *severe pain* and *mild daily* or *worse pain* were found to have good sensitivity and specificity. |
|  | Cadogan et al. (2004) | California, United States  16 LTC facilities  n=255 residents | - Cohort study - Compared LTC facilities in the upper and lower quartile for the QI | - Medical record analysis - Interviews with residents | The pain QI accurately differentiated the prevalence of pain between facilities, but higher prevalence of pain was associated with better performance in detection, assessment, treatment, and documentation of response to treatment. In the upper quartile group pain prevalence was 47% according to RAI-MDS data and according to interview data. In the lower quartile group, pain prevalence according to RAI-MDS data was significantly lower than the pain prevalence estimate on interview (9% versus 27%; χ^2^=5.62, p<.05). |
