# Supplementary material for: The Resident Assessment Instrument-Minimum Data Set 2.0 quality indicators: a systematic review
Source: BMC Health Serv Res. 2010 Jun 16;10:166. doi: 10.1186/1472-6963-10-166 (PMC2914032; doi:10.1186/1472-6963-10-166)
Supplement: Additional file 2 — Quality assessment of included studies. Quality assessment data from included studies [file 1472-6963-10-166-S2.DOC]

**Additional File 2 – Quality Assessment of Included Studies**

Adapted from Weightman AL, Mann MK, Sander L, Turley RL: Health Evidence Bulletins Wales. A systematic approach to identifying the evidence. Cardiff: Division of Information Services, University of Wales, College of Medicine; 2004.
